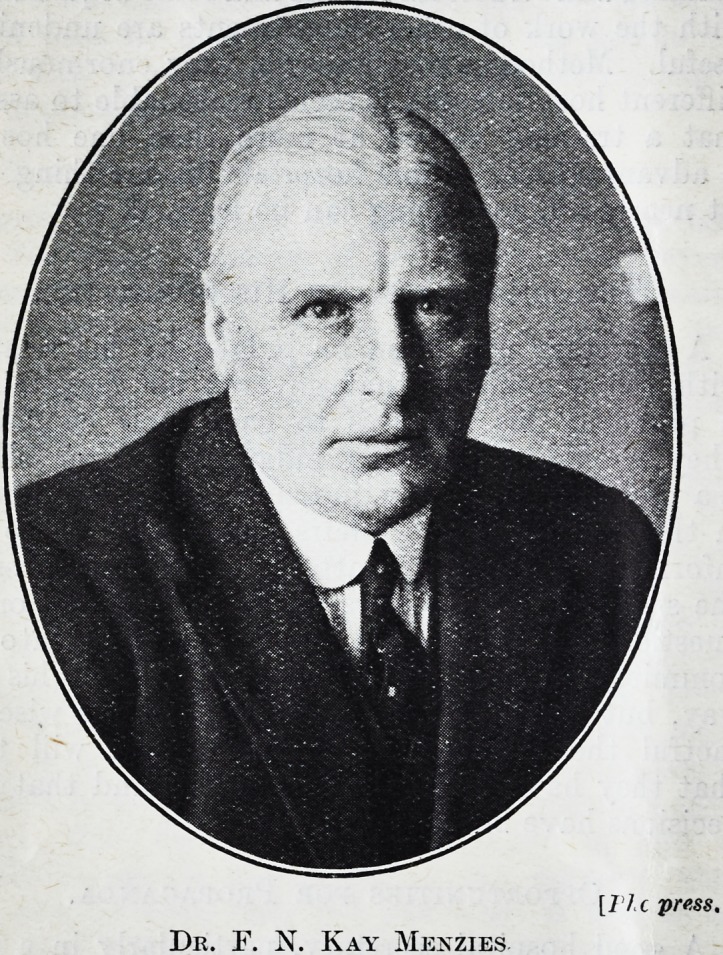# Red Cross Hospital Services: Dr. Kay Menzies as Administrator

**Published:** 1924-04

**Authors:** 


### RED CROSS HOSPITAL SERVICES.

### THE NEW DIRECTOR.

The Joint Council of the Order of St. John of Jerusalem and the British Red Cross Society has appointed Dr. F. N. Kay Menzies, F.R.C.P., to be Director of the Joint Council's Department of Hospital and Medical Services in succession to the late Sir Napier Burnett, K.B.E.

The appointment is one which will, undoubtedly, be welcomed by all concerned in the hospitals with which the Joint Council's activities are associated. Dr. Menzies has had a long and distinguished career in medicine, and possesses in high measure qualities and knowledge which fit him to deal with the varied

[*The press.*

Dr. F. N. Kay Menzies problems of hospital service. His experience in this direction has embraced, among other things, the work of House Physician to the Royal Infirmary, Edinburgh, and the Children's Hospital, Great Ormond Street, of Senior House Physician at the Hospital for Consumption, Brompton, and Assistant Lecturer in the Public Health Laboratories at University College, London.

As Principal Assistant Medical Officer in the Public Health Department of the London County Council, Dr. Menzies has been closely concerned with Public Health problems in their wider aspect, and he is in particular an authority on such subjects as tuberculosis, maternity, child welfare, etc. He is chairman of the Executive Committee of the Central Council for Infant and Child Welfare at Carnegie House, and a member of the Executive Committee of the Central Council for Rescue and Preventive Work For many years Dr. Menzies has been a member of the Council of the National Association for the Prevention of Tuberculosis, and was one of the founders of the Burrow Hill Colony for Tuberculous ex-Service Men at Frimley. He represented the British Red Cross Society at the Inter-Allied Conferences at Cannes (1919), Geneva (1920), and Copenhagen (1921), has been secretary of the Hygiene Section of the International Congress on Applied Chemistry (London), and is a Fellow of the Royal Society of Medicine and of the Royal Society of Arts.

His academic career took him to the Universities of Edinburgh, London, Vienna, and Berlin, and he has been a University examiner both in London and Liverpool. In a word, his equipment is such as to fit him admirably to fill a position in which breadth of view, familiarity with detail, insight and understanding must all be combined in a very special degree.

## Figures and Tables

**Figure f1:**